# Holistic Gaze Strategy to Categorize Facial Expression of Varying Intensities

**DOI:** 10.1371/journal.pone.0042585

**Published:** 2012-08-03

**Authors:** Kun Guo

**Affiliations:** School of Psychology, University of Lincoln, Lincoln, United Kingdom; University of Granada, Spain

## Abstract

Using faces representing exaggerated emotional expressions, recent behaviour and eye-tracking studies have suggested a dominant role of individual facial features in transmitting diagnostic cues for decoding facial expressions. Considering that in everyday life we frequently view low-intensity expressive faces in which local facial cues are more ambiguous, we probably need to combine expressive cues from more than one facial feature to reliably decode naturalistic facial affects. In this study we applied a morphing technique to systematically vary intensities of six basic facial expressions of emotion, and employed a self-paced expression categorization task to measure participants' categorization performance and associated gaze patterns. The analysis of pooled data from all expressions showed that increasing expression intensity would improve categorization accuracy, shorten reaction time and reduce number of fixations directed at faces. The proportion of fixations and viewing time directed at internal facial features (eyes, nose and mouth region), however, was not affected by varying levels of intensity. Further comparison between individual facial expressions revealed that although proportional gaze allocation at individual facial features was *quantitatively* modulated by the viewed expressions, the overall gaze distribution in face viewing was *qualitatively* similar across different facial expressions and different intensities. It seems that we adopt a holistic viewing strategy to extract expressive cues from all internal facial features in processing of naturalistic facial expressions.

## Introduction

Facial expressions of emotion display a wealth of visual information that we use to guide our social judgement and behaviour. The ability to recognize an individual's facial expression timely and to respond accordingly plays a crucial role in our social communication and even survival. Classical studies, such as those by Ekman and colleagues, have suggested six basic facial expressions, such as happy, sad, fear, anger, disgust and surprise, which can represent our typical emotional states and seem to have a universal meaning, regardless of the culture in which an individual is raised [1], [2] (see also [3]).

Given that our facial movements are controlled by the contraction and/or relaxation of facial muscles, Ekman and Friesen [2] developed a facial action coding system (FACS) to taxonomize human facial expressions. According to FACS, any anatomically possible facial expressions are associated with specific action units (movements of one or more muscles) and their temporal segments, and could be recognized and differentiated from each other. For instance, a typical happy face is correlated with raised inner eyebrows, cheek and upper lip, and tightened lower eyelid; and an angry expression comprises lowered eyebrows, eyes wide open with tightened lower lid, lips exposing teeth and stretched lip corners [2], [4]. It seems that each facial expression has one or more action units linked to key internal facial features such as eyes, nose and mouth. In other words, different facial features could provide expression-specific information in identifying different facial affects.

Findings from recent behaviour studies have supported this notion. When presenting parts of a face in isolation (e.g. through masking or ‘bubbles’ protocol in which participants perform an expression categorization task by viewing each face through a set of simultaneously presented, randomly allocated small Gaussian windows across the face), participants could rely on different facial parts to recognize basic expressions [5], [6]. The lower half of the face is more informative for labelling happiness, whereas the upper half is better for detecting fear and surprise. Furthermore, the basic facial expressions have minimal overlap in transmitted facial information and different facial features can provide diagnostic information in recognizing different expressions. For example, the eyes and mouth region transmit crucial cues for detecting angry and happy expressions, respectively [5]. Given these findings in FACS and diagnostic facial regions [2], [4], [5], it is plausible that in a situation of face exploration when free eye movements are permitted, the gaze distribution to the eye, nose and mouth regions could be systematically influenced by the viewed facial expressions.

To date, several eye tracking studies have examined the role of fixations in extracting diagnostic information to recognize different facial expressions. Typically during a self-paced expression categorization task, the participants tend to direct longer viewing time and/or more fixations towards the eye rather than the mouth or nose region [3], [7]–[9]. The dwell time at the eyes could be increased when viewing some negative expressions, such as fear and anger [8]. One recent study further examined how individual facial expression affected gaze allocation at the eyes and mouth region, and found that participants fixated more to the eyes in the sad or angry face, but to the mouth in the happy face. The eyes and mouth region in the fearful and neutral faces, on the other hand, tended to attract similar amount of fixations [10]. It seems that people do look at local facial regions that are most characteristic for each facial expression.

Although the above discussed behavioural and eye-tracking studies have suggested the critical role of individual facial features in transmitting diagnostic cues for decoding facial expressions, the generalisation of the finding could be limited by one methodological problem. That is, the expressive faces used in those studies tended to pose an exaggerated configuration of muscle movements for each emotion category or represent peak emotional expressions. In our daily life, however, we see less intense expressions more frequently than intense ones. Behavioural studies have shown that facial affects displayed in low-intensity would significantly increase our difficulty to interpret subtle expressions [11], [12]. Considering that expressive cues from individual facial features would be ambiguous in low-intensity, we probably need to rely more on configural information and/or combine expressive cues from more than one facial feature to reliably decode facial affect. Given perceptually people tend to recognize face through a holistic process of perceiving relations among individual facial features and integrating all features into an individual representation of the face as a whole [13], lowering expression intensity may promote a similar ‘holistic’ face-viewing behaviour (i.e. scanning all the key internal facial features to extract expressive cues from the whole face rather than just from single part of the face) which could be more close to the gaze strategy we use in everyday life and facilitate the holistic face processing. To investigate this possibility, in this study we applied a morphing technique to create blends between neutral and expressive faces which simulate facial muscle movements in a linear manner. We systematically measured participants' behavioural performance to differentiate the six basic facial expressions from neutral, and compared the associated gaze patterns to examine the role of fixations in processing expressions at varying levels of intensity.

## Materials and Methods

To control potential gender difference in expression categorization performance and associated gaze pattern [9], only female participants were recruited. In total, 28 female undergraduate students, age ranging from 18 to 21 years old with the mean of 19.43±0.19 (Mean±SEM), volunteered to participate in the study. All participants had normal or corrected-to-normal visual acuity. The Ethical Committee in School of Psychology, University of Lincoln approved this study. Written informed consent was obtained from each participant, and all procedures complied with the British Psychological Society “Code of Ethics and Conduct”, and with the World Medical Association Helsinki Declaration as revised in October 2008.

Digitized grey scale face images in full frontal view were presented through a ViSaGe graphics system (Cambridge Research Systems, UK) and displayed on a non-interlaced gamma-corrected colour monitor (30 cd/m^2^ background luminance, 100 Hz frame rate, Mitsubishi Diamond Pro 2070SB) with the resolution of 1024×768 pixels. At a viewing distance of 57 cm the monitor subtended a visual angle of 40×30°.

**Figure 1:**
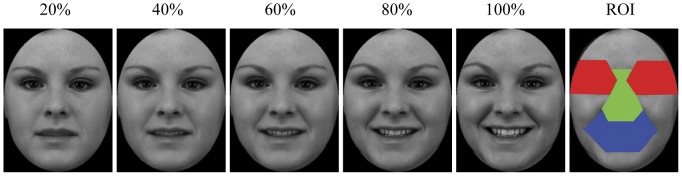
Examples of happy expression at varying intensity levels. The face labelled as ROI (region of interest) shows an example of the facial regions that were used in the eye-tracking analyses. The red, green and blue areas represent the eyes, nose and mouth regions, respectively. The copyrighter holder of the image has given written informed consent, as outlined in the PLoS consent form, to publication of their photograph.

Twenty-eight Western Caucasian face images, consisting of two female and two male models, were selected from Karolinska Directed Emotional Faces [14]. Each of these models posed one neutral and six high-intensity facial expressions (happiness, sadness, fear, anger, disgust and surprise). Although they may have real-world limitations and categorization performance for some expressions could be subject to culture influence [3], these well-controlled face images were chosen for their comparability and universality in transmitting facial expression signals, at least for our observer group (Western Caucasian adults). The faces were processed in Adobe Photoshop to remove external facial features (e.g. hair) and to ensure a homogenous grey background, same face size and brightness. For each of the six expressions of each model, we then used Morpheus Photo Morpher to create 5 levels of intensity ranging from 20 to 100% with 20% increments by morphing the emotional face with the neutral face. As a result, 120 expressive face images were generated for the testing session (6 expressions×5 intensities×4 models, see Fig. 1 for examples). These images were gamma-corrected and displayed once in a random order at the centre of the screen with a resolution of 420×600 pixels (15×22°).

All of our participants were aware of universal facial expressions. Before the recording, they were shown a PowerPoint presentation containing one male and one female models posing happiness, sadness, fear, anger, disgust and surprise expressions (sampled from Pictures of Facial Affect), and were asked to label each facial expression as carefully as possible without time constraint. All of them could recognize these facial expressions or agreed with the classification proposed by Ekman and Friesen [1].

During the self-paced experiments the participants sat in a chair with their head restrained by a chin rest, and viewed the display binocularly. To calibrate eye movement signals, a small red fixation point (FP, 0.3° diameter, 15 cd/m^2^ luminance) was displayed randomly at one of 9 positions (3×3 matrix) across the monitor. The distance between adjacent FP positions was 10°. The participant was instructed to follow the FP and maintain fixation for 1 sec. After the calibration procedure, the participant pressed the response box to initiate a trial. The trial was started with a FP displayed on the centre of the monitor. If the participant maintained fixation for 1 sec, the FP disappeared and a face image was presented. During the self-paced, free-viewing presentation, the participant was instructed to “categorize this facial expression as accurately and as quickly as possible”, and to respond by pressing a button on the response box (for collecting reaction time data) followed by a verbal report of the perceived facial expression (6-alternative forced-choice: happiness, sadness, fear, anger, disgust and surprise). No reinforcement was given during this procedure.

Horizontal and vertical eye positions were measured using a Video Eyetracker Toolbox with 250 Hz sampling frequency and up to 0.25° accuracy (Cambridge Research Systems). The software developed in Matlab computed horizontal and vertical eye displacement signals as a function of time to determine eye velocity and position. Fixation locations were then extracted from the raw eye tracking data using velocity (less than 0.2° eye displacement at a velocity of less than 20°/s) and duration (greater than 50 ms) criteria [15].

While determining fixation allocation within key internal facial features (i.e. eyes, nose and mouth), we adopted consistent criteria to define boundaries between local facial features for different faces [16] to ensure equal size of individual internal feature across faces of different expressions and intensities from the same model. Specifically, the ‘eye’ region included the eyes, eyelids, and eyebrows; the ‘nose’ or ‘mouth’ region consisted of main body of the nose (glabella, nasion, tip-defining points, alar-sidewall, and supra-alar crease) or mouth and immediate surrounding area (up to 0.5° visual angle). The division line between the ‘mouth’ and ‘nose’ regions was the midline between upper lip and the bottom of the nose (Fig. 1). Each fixation was then characterised by its location among feature regions and its time of onset relative to the start of the trial, and the number of fixations directed at each feature was normalized to the total number of fixations sampled in that trial. As we required the participants to fixate a central FP prior to image presentation, the ‘first’ recorded fixation following the face appearance was likely to be the artefact of this central FP procedure and was hence removed from further analysis.

## Results

### Analysis of pooled expressions

**Figure 2:**
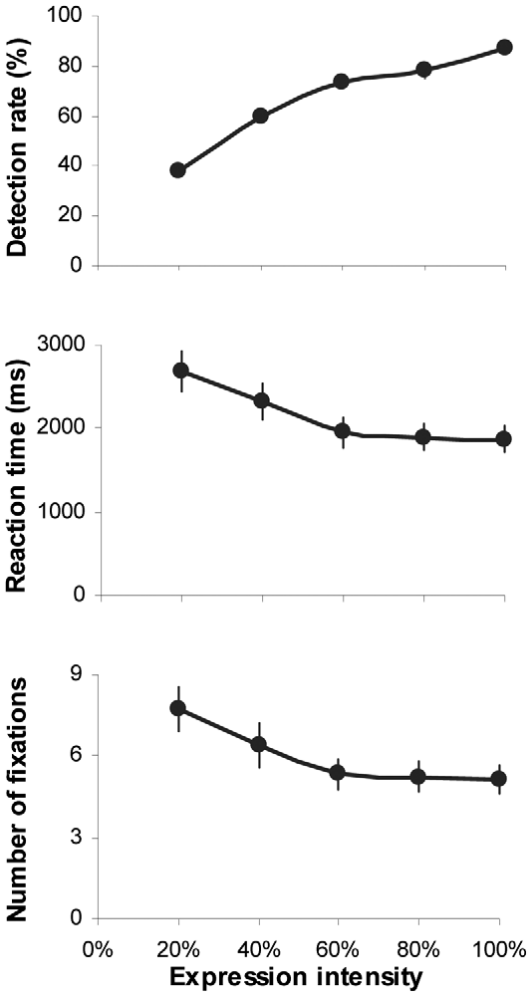
Mean accuracy, reaction time and number of fixations allocated at the faces for expression categorisation as a function of intensity. Error bars represent SEM.

We first examined to what extent the expression intensity would affect participants' overall task performance in categorizing facial expressions. Three repeated-measures analysis of variance (ANOVA) with expression intensity as the independent variable, percentage of correct expression identification, reaction time and averaged number of fixations directed at each face as the dependent variables were conducted. The analysis demonstrated that intensifying expression intensity would significantly increase the accuracy of expression categorization (*F*(4,108) = 117.56, *p*<0.001, *η_p_^2^* = 0.81), shorten the reaction time (*F*(4,108) = 36.46, *p*<0.001, *η_p_^2^* = 0.58) and reduce the number of fixations allocated at the faces (*F*(4,108) = 35.9, *p*<0.001, *η_p_^2^* = 0.57; Fig. 2). This facilitation influence was the most evident for the intensity increase up to 60% (Bonferroni correction for multiple comparisons, *p*<0.01). Higher (>60%) intensity had no further impact on the reaction time and fixation numbers (*p*>0.61).

**Figure 3:**
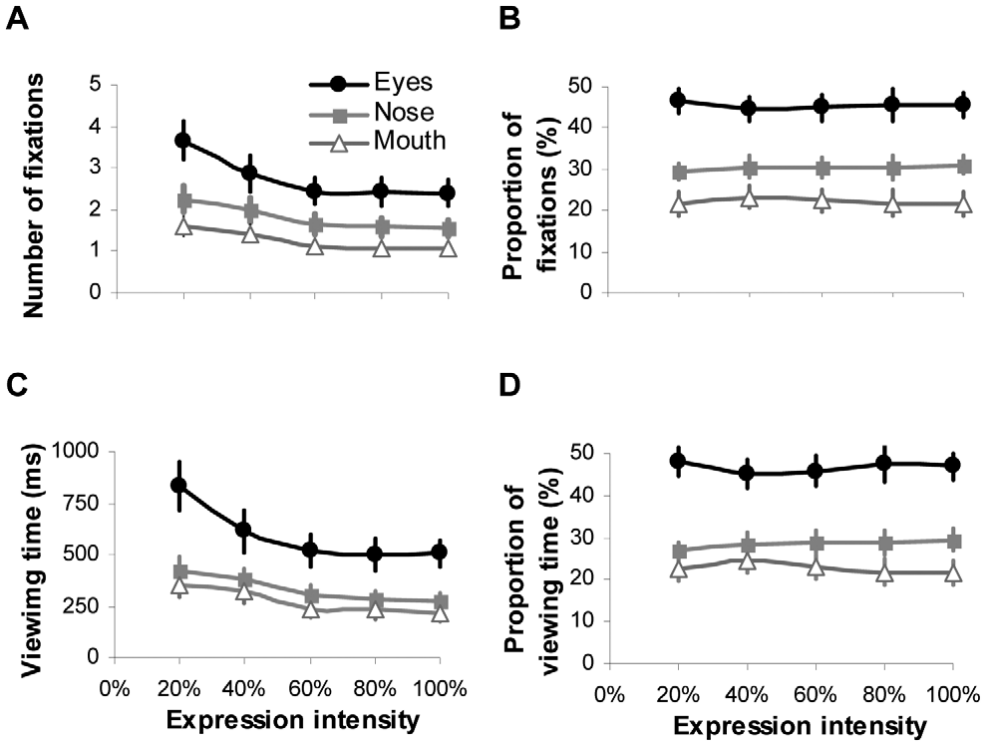
Number of fixations (A) and viewing time (C), and normalised proportion of fixations (B) and viewing time (D) directed at the eyes, nose and mouth regions during the task of categorizing facial expressions with varying intensities. Error bars represent SEM.

Considering that during the expression categorization, the vast majority of fixations (98%±0.1 of overall fixations per trial) and viewing time (98%±0.2 of total face viewing time per trial) was allocated at key internal facial features (i.e. eyes, nose and mouth), we then conducted 5 (intensity)×3 (facial feature) ANOVAs to examine how the fixation and viewing time allocated at key facial features were modulated by varying expression intensities. Overall, the pattern of gaze distribution in the task of expression categorization was similar to those reported in the task of free-viewing and identity recognition [16]–[19]. Among internal facial features, the eyes tended to attract the highest numbers of fixations and the longest viewing time, followed by the nose and then the mouth regardless of expression intensities (fixation: *F*(2,54) = 18.26, *p*<0.001, *η_p_^2^* = 0.4; viewing time: *F*(2,54) = 15.11, *p*<0.001, *η_p_^2^* = 0.36; Fig. 3A and 3C). Increasing expression intensity significantly reduced the amount of fixations and viewing time directed at all facial features (fixation: *F*(4,108) = 35.2, *p*<0.001, *η_p_^2^* = 0.57; viewing time: *F*(4,108) = 35.7, *p*<0.001, *η_p_^2^* = 0.57). However once the intensity has reached 60%, further increase had no impact on the fixations/viewing time towards individual facial features (Bonferroni correction for multiple comparisons, *p*>0.5).

Analysis of the normalised gaze distribution showed that the facial features also had a significant impact on the proportional fixation (*F*(2,54) = 20.6, *p*<0.001, *η_p_^2^* = 0.43; Fig. 3B) and viewing time distribution (*F*(2,54) = 19.27, *p*<0.001, *η_p_^2^* = 0.42; Fig. 3D). The expression intensity, on the other hand, did not affect proportion of fixations (*F*(4,108) = 0.64, *p* = 0.63, *η_p_^2^* = 0.02) and viewing time (*F*(4,108) = 0.56, *p* = 0.69, *η_p_^2^* = 0.02) allocated at eyes, nose or mouth region. It seems that when categorizing facial expression of varying intensities, our participants adopted a consistent gaze strategy to extract expressive cues from eyes, nose and mouth regions. Although higher expression intensity would reduce the absolute amount of fixations/viewing time directed at local facial features, the proportional distribution of fixations/viewing time among local features was unchanged.

### Analysis of individual expressions

Previous studies have demonstrated different perceptual sensitivities in recognizing different facial expressions. Specifically, people often have the most accurate and fastest identification performance for happiness, but are least accurate in recognizing fearful (or anxious) expressions [20]–[22]. To examine how expression intensity would affect participants' behavioural responses in categorizing individual facial expressions, we conducted 5 (intensity)×6 (expression) ANOVAs with categorization accuracy, reaction time and number of fixations per face as the dependent variables.

**Figure 4:**
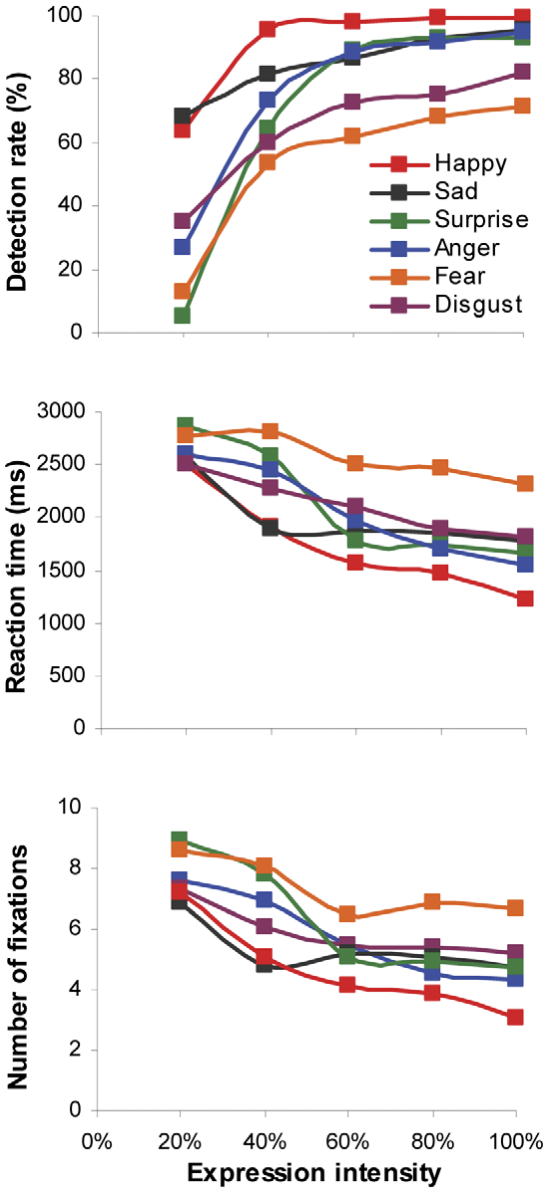
Mean accuracy, reaction time and number of fixations allocated at the faces for expression categorisation as a function of intensity. Different curve represents different facial expression of emotions.

Although increasing expression intensity would improve categorization accuracy (*F*(4,108) = 438.8, *p*<0.001, *η_p_^2^* = 0.94; Fig. 4), reduce reaction time (*F*(4,108) = 42.16, *p*<0.001, *η_p_^2^* = 0.61) and number of fixations in face viewing (*F*(4,108) = 38.92, *p*<0.001, *η_p_^2^* = 0.59), the degree of its impact varied with individual facial expressions (categorization accuracy: *F*(5,135) = 36.3, *p*<0.001, *η_p_^2^* = 0.57; reaction time: *F*(5,135) = 21.84, *p*<0.001, *η_p_^2^* = 0.45; fixation numbers: *F*(5,135) = 19.26, *p*<0.001, *η_p_^2^* = 0.42). Among six tested expressions, participants tended to direct the least amount of time and fixations to view happy faces but showed the highest detection accuracy; they used the longest time and the most number of fixations to view fearful faces but showed the poorest categorization accuracy. Such behavioural response to happy and fearful expressions started to differentiate at the lowest expression intensity (20%) and lasted through the whole testing range of intensities (Bonferroni correction for multiple comparisons, all *ps*<0.01). Detailed comparisons between individual expressions further revealed that the participants were also sensitive to sad expression displayed at lower intensities. The detection rates, required viewing time and number of fixations were similar to those needed for recognizing happy expression (all *ps*>0.05). Furthermore, they demonstrated indistinguishable behaviour performance in classifying surprise, disgust and anger expressions (all *ps*>0.05).

Given relatively poor categorization accuracy for low-intensity expressive faces (Fig. 4), we computed confusion matrices to illustrate which expressions were mistaken for others and to examine whether there were systematic biases in categorizing different expression intensities. As shown in Table 1, subtle (20% intensity) happy, anger, surprise, disgust and fear expressions were most likely mislabelled as sad expression (Bonferroni correction for multiple comparisons, all *ps*<0.01). Low-intensity surprised and fearful faces were also often perceived as happy faces (all *ps*<0.01). No systematic mis-categorization bias was observed for sad expression and medium/high-intensity (≥40%) happy, anger and surprise expressions (all *ps*>0.05). On the other hand, among wrongly classified expression with 40% or higher intensity, fear was often confused with surprise expression, and disgust was likely to be mislabelled as anger or sad expression.

**Table 1:** Confusion matrices of expression categorization: percentage of participants selecting the expression labels, averaged across the stimulus set and participants.

Displayed expression		Categorized expression (%)					
	Intensity	Happy	Sad	Anger	Surprise	Disgust	Fear
Happy	20%	**62.50**	15.18	8.04	6.25	3.57	4.46
	40%	**95.54**	0.89	0.89	0.89	0.00	1.79
	60%	**99.11**	0.00	0.00	0.00	0.00	0.89
	80%	**99.11**	0.89	0.00	0.00	0.00	0.00
	100%	**99.11**	0.00	0.00	0.89	0.00	0.00
Sad	20%	8.04	**67.86**	7.14	5.36	6.25	5.36
	40%	3.57	**81.25**	4.46	1.79	6.25	2.68
	60%	0.00	**86.61**	1.79	0.00	6.25	5.36
	80%	0.00	**92.86**	0.89	0.89	1.79	3.54
	100%	0.00	**95.54**	0.00	0.89	0.89	2.68
Anger	20%	9.82	38.39	**26.79**	8.93	10.71	5.36
	40%	1.79	12.50	**75.00**	3.57	5.36	1.79
	60%	0.00	2.68	**88.39**	1.79	4.46	2.68
	80%	0.00	2.68	**91.96**	1.79	2.68	0.89
	100%	0.00	1.79	**94.64**	1.79	0.89	0.89
Surprise	20%	24.11	42.86	8.93	**5.36**	9.82	8.93
	40%	8.04	15.18	0.89	**64.29**	1.79	9.82
	60%	4.46	1.79	0.00	**89.29**	0.89	3.57
	80%	0.89	0.00	0.00	**93.75**	0.89	4.46
	100%	1.79	0.89	0.00	**92.86**	0.00	4.46
Disgust	20%	10.71	28.57	15.18	5.36	**34.82**	5.36
	40%	4.46	16.07	14.29	1.79	**59.82**	3.57
	60%	0.00	11.61	12.50	0.89	**72.32**	2.68
	80%	2.68	8.04	13.39	0.00	**75.00**	0.89
	100%	0.00	6.25	10.71	0.00	**82.14**	0.89
Fear	20%	18.75	39.29	10.71	9.82	8.93	**12.50**
	40%	4.46	8.04	2.68	22.32	8.93	**53.57**
	60%	0.89	2.68	3.57	23.21	8.04	**61.61**
	80%	0.89	1.79	2.68	17.86	8.93	**67.86**
	100%	0.00	1.79	1.79	16.07	8.93	**71.73**

**Figure 5:**
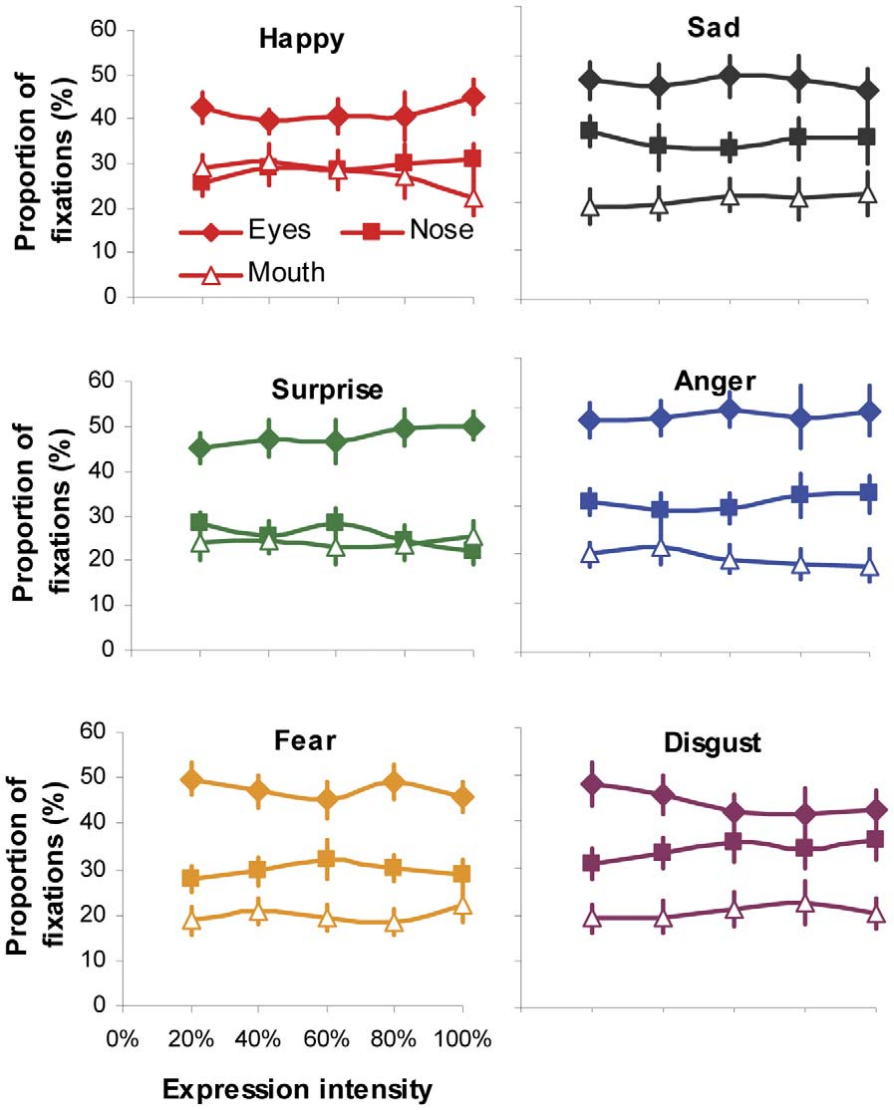
Normalised proportion of fixations directed at the eyes, nose and mouth regions when categorizing different facial expressions with varying intensity. Error bars represent SEM.

**Figure 6:**
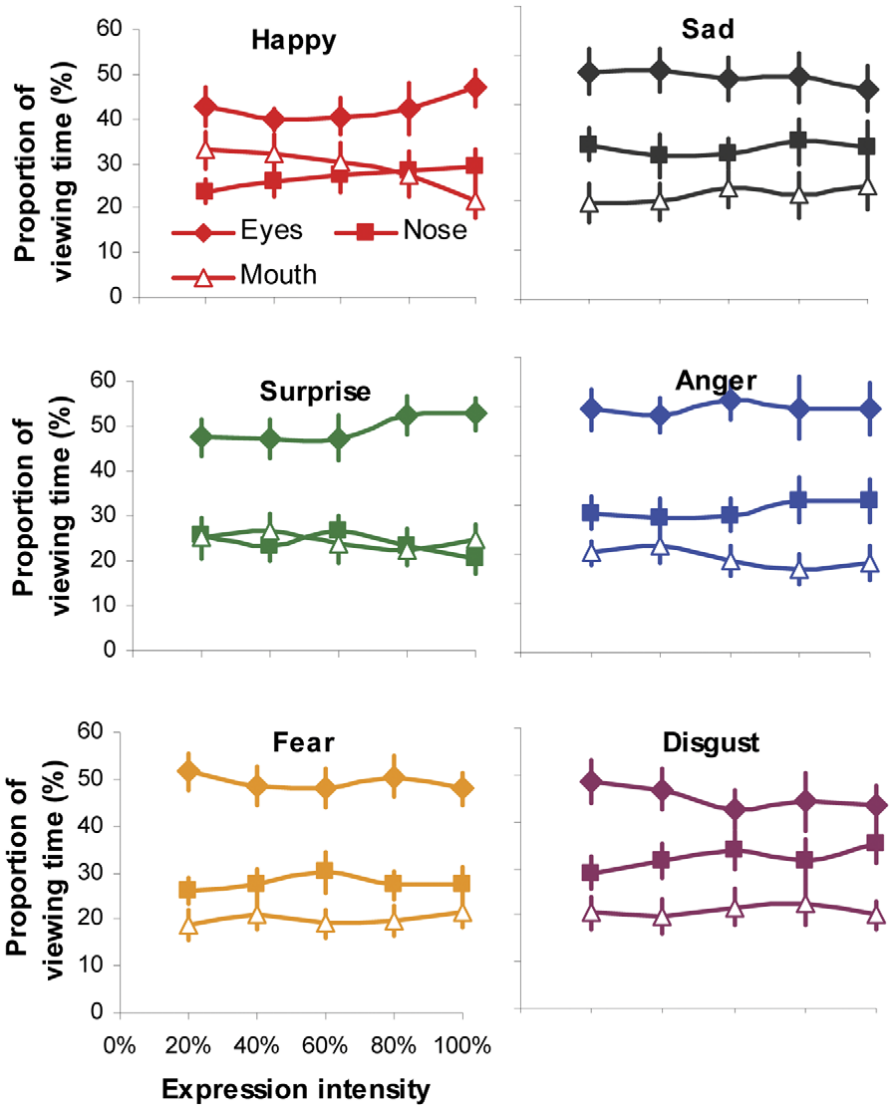
Normalised proportion of viewing time directed at the eyes, nose and mouth regions when categorizing different facial expressions with varying intensity. Error bars represent SEM.

We then run 5 (intensity)×6 (expression)×3 (facial region) ANOVA to examine whether the expression intensity would affect gaze distribution at local facial regions for individual viewed expressions. The significant main effect of facial region on fixation (*F*(2,54) = 23.14, *p*<0.001, *η_p_^2^* = 0.46; Fig. 5) and viewing time distribution (*F*(2,54) = 21.81, *p*<0.001, *η_p_^2^* = 0.45; Fig. 6) indicated that the eye region attracted the highest proportion of fixation and viewing time in face-exploring irrespective of the viewed facial expression and its intensities. The non-significant main effect of expression (fixation: *F*(5,135) = 1.47, *p* = 0.2, *η_p_^2^* = 0.05; viewing time: *F*(5,135) = 1.37, *p* = 0.24, *η_p_^2^* = 0.05) and intensity (fixation: *F*(4,108) = 0.55, *p* = 0.7, *η_p_^2^* = 0.02; viewing time: *F*(4,108) = 0.52, *p* = 0.72, *η_p_^2^* = 0.02), on the other hand, indicated that the participants tended to direct the same proportion of fixation and viewing time to individual facial features (eyes, nose or mouth region) during the process of categorizing a specific facial expression regardless of its intensities. This was further supported by non-significant interaction observed between expression intensity and facial expression or facial regions (all *ps*>0.42).

**Figure 7:**
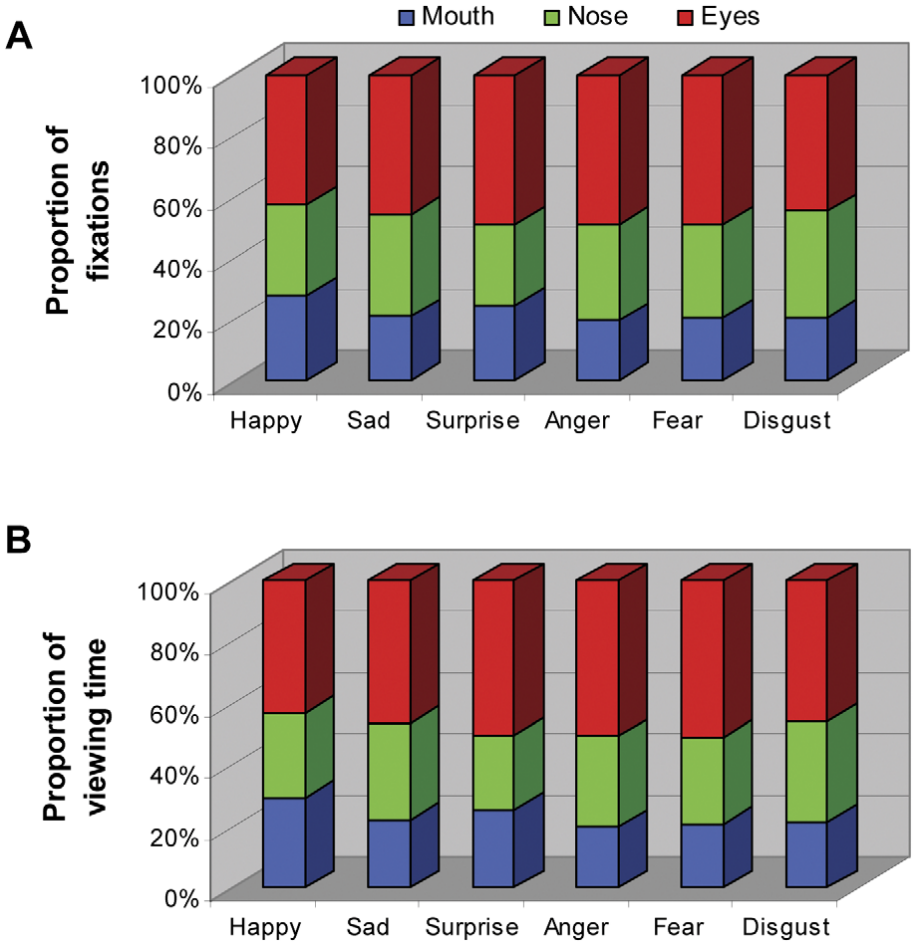
Proportion of fixations (A) and viewing time (B) directed at the eyes, nose and mouth regions when categorizing different facial expressions. For each expression, data sampled from different intensities were collapsed together.

The significant interaction between facial expression and facial region (fixation: *F*(10,270) = 6.83, *p*<0.001, *η_p_^2^* = 0.2; viewing time: *F*(10,270) = 7.88, *p*<0.001, *η_p_^2^* = 0.23), however, suggested that quantitatively the proportion of fixation (Fig. 7A) and viewing time (Fig. 7B) directed at the same facial feature was expression-specific. The detailed pairwise comparison further showed that for the mouth region, the mouth in happy face tended to attract the largest proportion of fixation and viewing time, followed by the mouth in surprised face (all *ps*<0.01); the mouth in the faces of other expressions drew the same amount of attention from our participants (all *ps*>0.05). For the nose region, the participants directed the largest amount of fixation and viewing time at the nose in disgust and sad faces, and the least amount at the nose in surprised face (all *ps*<0.05); the nose in happy, fear and angry faces attracted indistinguishable amount of attention. As for the eye region, the eyes in happy face was the least viewed, followed by the eyes in sad or disgust face; the eyes in angry, fearful or surprised face, on the other hand, was the most frequently or the longest viewed facial feature (all *ps*<0.05). Taken together, it seems that when categorizing facial expressions, the participants tended to extract different amount of information from the same facial feature in different type of expressive faces.

**Figure 8:**
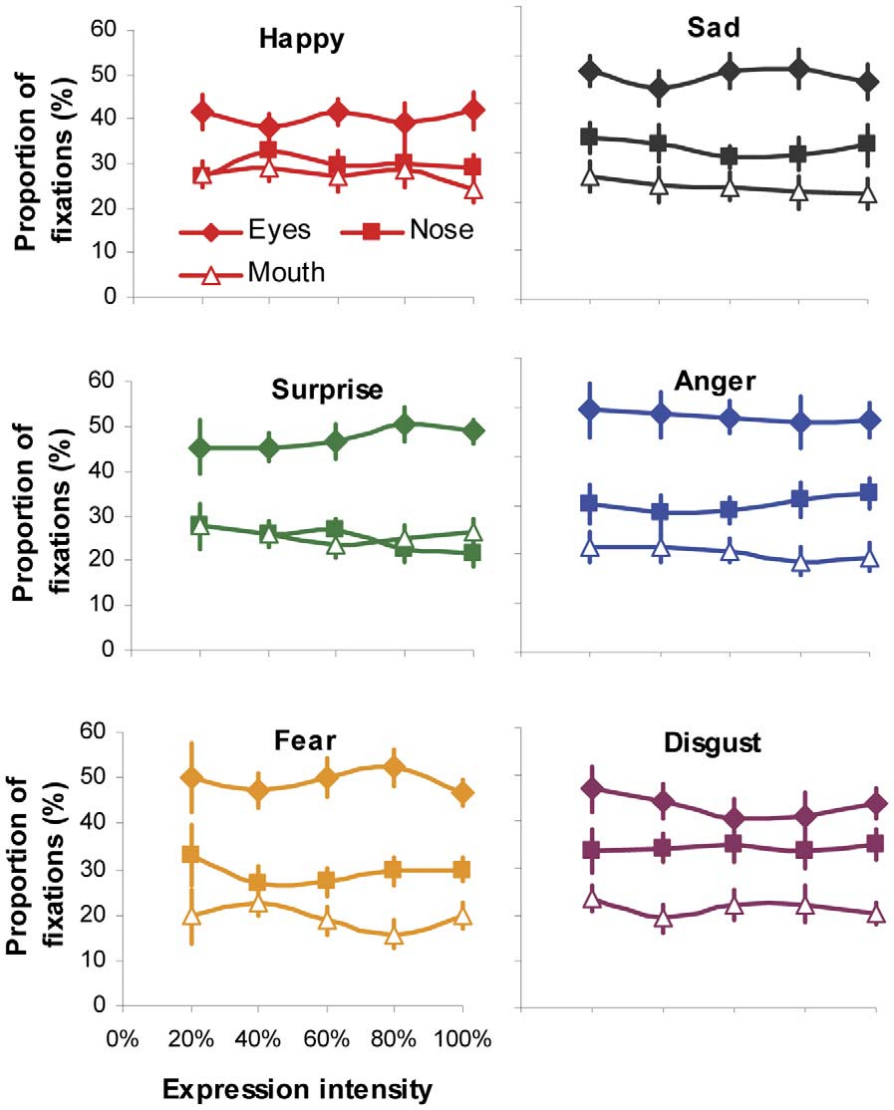
Normalised proportion of fixations directed at the eyes, nose and mouth regions when categorizing different facial expressions with varying intensity. Values presented in the graph were only collected from trials leading to correct expression identification. Error bars represent SEM.

It should be noted that gaze distribution shown in Figure 5 and 6 was analyzed by using data from all the trials rather than just from trials with correct expression identification. In other words, the data was grouped according to viewed rather than perceived expressions. Hence the observed gaze behaviour was more likely to be associated with the viewing of facial expressions, but not necessarily linked with the categorization performance. To examine to what extent the categorization accuracy affect gaze behaviour, we re-analyzed fixation distribution at local facial regions for individual expressions and intensities using data only from accurate categorization trials (Fig. 8). Similar as those observed in Figure 5, 5 (intensity)×6 (expression)×3 (facial region) ANOVA revealed non-significant main effect of expression or intensity, and non-significant interaction between intensity and facial expression or facial region (all *ps*>0.64). The same conclusion was found for viewing time distribution analysis. It seems that while making correct identification to a specific facial expression, the participants directed the same proportion of fixation and viewing time to individual facial features (eyes, nose or mouth region) regardless of expression intensities. The close similarity of fixation distribution between Figure 5 and Figure 8 was not unexpected, given higher categorization accuracy (>75%) for majority of the tested expressions and intensities (Table 1). However, as some low-intensity expressions (e.g. 20% surprised or fearful faces) had poor recognition performance and hence very few valid trials, this part of result should be treated with cautious.

## Discussion

Although we have daily encounters with many expressive faces, our ability in recognizing facial expressions of emotion is often affected by the type of displayed expression and its intensity. In general, our categorization accuracy is expression-dependent and increases with the increasing expression intensity [12]. At the peak intensity, people often have the most accurate and fastest identification performance for happiness (sometimes also for surprise expression), but are least accurate in recognizing fearful (or anxious) expressions [9], [20]–[22]. A similar effect of facial expression and its intensity on categorization performance was also observed in this study. Increasing expression intensity significantly improved categorization accuracy, shortened reaction time and reduced number of fixations directed at the faces (Fig. 2). The degree of such facilitatory effect, however, varied across six tested expressions. Happy faces attracted the highest identification accuracy but the least amount of viewing time and fixations; Fearful faces, in contrast, had the poorest categorization accuracy but needed the longest viewing time and the most number of fixations. No significant difference in behaviour performance was observed in classifying surprise, disgust and anger expressions (Fig. 4). Although it is still unclear why different facial affects are associated with different categorization performance, our prior experience in processing different expressions may play an important role [9].

In addition to superior categorization performance for happy and sad expressions displayed at low intensities (Fig. 4), we also observed clear categorization biases toward these two expressions. When confronted with low-intensity surprised or fearful faces, the participants were more likely to label the expression as sad, followed by happy. Subtle anger and disgust expressions were also often confused with sad expression (Table 1). Probably because surprise, fear, anger and disgust are frequently expressed with high intensities whereas subtle sadness and happiness are relatively common emotional expressions in everyday social interactions, we are perceptually more sensitive to recognize these two facial affects [12] and are more inclined to label low-intensity ambiguous expressions (such as subtle signals of surprise or fear) as sad or happy. For medium and high-intensity facial expressions, there was asymmetric pattern of confusion between fear and surprise (i.e. fear was consistently mistaken for surprise but not vice versa), and between disgust and anger. These categorization biases may be related to the shared muscle action units between confused expressions [2], frequency of prior exposure to these expressions [12], and differences among expressions in salience and signal value [23]. The exact cause of expression categorization bias and its role in social interaction is an interesting and important question for future research to address.

By presenting part of an intensified expressive face in isolation (i.e. through masking or ‘bubbles’ protocol), earlier studies have observed that participants could solely rely on different facial parts to recognize some facial expressions [5]. For instance, the lower half of the face is more informative for labelling happy expression, whereas the upper half is better for detecting fear and surprise. However in our testing situation when the whole face with varying expressive intensity was presented and free eye movements were permitted, the participants tended to adopt a more ‘holistic’ approach. They were likely to scan all the key internal facial features and the pattern of gaze distribution among these features was *qualitatively* identical, irrespective of facial expression and its intensities. Specifically, the eyes always attracted the highest proportion of fixations and viewing time, followed by the nose and mouth (Fig. 3). Although increasing expression intensity would enable the participants to direct less fixations/viewing time at local facial features to classify expressions, the proportional distribution of fixations/viewing time among local features was unchanged across all the tested intensities either for expressive faces as a whole (Fig. 3) or for individual type of facial expressions (Fig. 5, 6). In other words, our participants adopted a constant and holistic gaze strategy to extract expressive cues from eyes, nose and mouth regions while performing facial expression categorization task.

This finding is in agreement with the hypothesis of holistic representation in processing facial expressions, also supported by some electrophysiological and behavioural studies. For instance, while performing emotion categorization task, a substantial proportion of neurons in human amygdale, the brain region playing a central role in processing emotions, showed preferential responses to the whole expressive faces as opposed to individual facial features. Furthermore, the neural responses to facial parts were not predictive of the responses to the whole faces [24]. Behaviourally, participants were significantly slower and less accurate to classify facial expressions shown in either top or bottom half of the composite faces (e.g. aligning the top half of one expression with the bottom half of another) than the non-composite faces (even two halves are mis-aligned), suggesting facial expressions would be more effectively processed by integrating local expressive cues from both top and bottom half of the faces [25]. Taken together, it seems that this holistic representation is manifested through different stages of facial emotion processing, from extracting local expressive facial cues (e.g. gaze allocation), to processing acquired facial information (e.g. amygdale responses), and then to behaviourally categorizing perceived facial expressions.

It should be emphasized that our observation is not inconsistent with the previous findings of different local facial features transmitting different diagnostic expressive cues. Using expressive faces at peak intensity, the ‘bubble’ studies have suggested that the mouth region transmits critical information for detecting happiness expression, nose with its surrounding area contains cues for disgust recognition, and the eyes are diagnostic for perceiving anger and fear [5]. In the present study, the viewed facial expressions with varying intensity also quantitatively affected the proportion of fixations/viewing time directed at the eyes, nose or mouth region. Compared with the same local region in different facial expressions, the participants looked more often at the mouth region in happy faces, at the nose region in disgust and sad faces, and at the eyes in angry, fearful and surprise faces (Fig. 7). Another recent eye-tracking study [10] compared gaze allocation at eyes and mouth region when participants examined different facial expressions displayed at peak intensity, and found the similar expression-dependent gaze distribution. It seems that humans tend to look at the most characteristic facial region when perceiving emotional faces with not just peak intensity but also medium or lower intensities (>20% in this case).

However, as expressive faces often reflect intricate combinations of facial feature movements, the expressive cue from a single facial feature is often ambiguous and unreliable for accurate categorization [3]. For instance, there is considerable variability in local facial regions across different individuals and different situations to express some facial expressions, such as angry (frowning, outer brow raised, visible teeth, lower lip depressed, lips tightly closed, etc.) [4]. Such ambiguity of expressive cues from local facial feature would be more evident for expressive faces at lower intensities. It is worth pointing out that in our study participants rarely labelled an expression (even at peak intensity) after fixating only at single characteristic facial region. Instead they often analyzed facial information sampled from the diagnostic region (e.g. mouth in happy face) in conjunction with those from other key internal facial features (e.g. eyes and nose region) before labelling the expression. Furthermore, the characteristic facial region of individual expression is unlikely to dominate or determine the overall gaze distribution within a face. For instance, the mouth region is the characteristic feature for happy expression, but the eyes in happy face still attract the largest proportion of fixations and viewing time. Interestingly, this pattern of disproportionate share of gaze at the eyes is not restricted to expression categorization task [3], [10], but has also been observed in other cognitive tasks, such as free viewing, face learning, face familiarity or identity judgement [16]–[19], [26], suggesting a crucial role of the eyes in transmitting various facial information and possibly a generic ‘built-in’ scanning strategy in our brain for general face processing [27].

To conclude, we demonstrated that different facial expressions of emotion with varying intensities could systematically influence our recognition performance. In general the behavioural response was expression-dependent, and increasing expression intensity would significantly improve categorization accuracy, shorten reaction time and reduce number of fixations directed at faces. Although proportionally gaze allocation at individual facial region (i.e. eyes, nose and mouth) was quantitatively modulated by the viewed expressions, the qualitative gaze pattern in face viewing was similar across different facial expressions and different intensities. It seems that humans employ a ‘holistic’ viewing strategy to categorize facial affects. Regardless of expression category and displayed intensity, we tend to scan all key facial features to reliably label expressions, and allocate most of the gaze at the eye region, followed by the nose and the mouth regions.
